# Meningitis outbreak investigation in Nkoranza South Municipality in Brong Ahafo Region, Ghana, February, 2016

**DOI:** 10.11604/pamj.supp.2018.30.1.15261

**Published:** 2018-05-16

**Authors:** Ernest Konadu Asiedu, Kofi Mensah Nyarko, Ernest Kenu, Edwin Andrew Afari, Joseph Asamoah Frimpong, Meeyoung Mattie Park, Scott JN McNabb, Florence Nzilanye Iddrisah

**Affiliations:** 1 Ghana Field Epidemiology and Laboratory Training Program, Ghana; 2 School of Public Health, University of Ghana, Ghana; 3 National Catholic Health Service, Accra, Ghana; 4 African Field Epidemiology Network, Accra, Ghana; 5 Rollins School of Public Health Emory University, Atlanta, USA

**Keywords:** Meningitis, outbreak investigation, Ghana, data analysis, epidemiology

## Abstract

The occurrence of communicable diseases highlights the need to have well-trained field epidemiologists at the forefront in the fight against these diseases, especially during an outbreak. Training for outbreak investigation is most effective when participants can develop their competencies in a practical exercise. This is a simulation of the steps in meningitis outbreak investigation conducted in Ghana in February 2016 by Ghana Field Epidemiology Training Programme (FELTP) residents and the public health technical team of the Nkoranza South Municipality as a field epidemiologist. This case study is suited to reinforce principles and skills already covered in a lecture or in background reading by providing a practical training beyond the scope of theoretical learning. It is primarily intended for training novice public health practitioners who should be able to complete the exercises in 3 hours.

## How to use this Study

**General instructions:** ideally, 1 to 2 instructors facilitate the case study for 13 to 20 students in a classroom or conference room. The instructor should direct participants to read a paragraph out loud, going around the room to give each participant a chance to read. When the participant reads a question, the instructor directs all participants to perform calculations, construct graphs, or engage in discussions. The instructor may split the class to play different roles or take different sides in answering a question. As a result, participants learn from each other, not just from the instructors. Specific instructor’s notes are included with each question in the instructor’s version of this case study.

**Audience:** residents in Ghana Field Epidemiology Training Programs (Ghana-FELTP) and other Field Epidemiology and Laboratory Training Programs (FELTPs) and Field Epidemiology Training Programs (FETPs) who are interested in this topic.

**Prerequisites:** before using this case study, case study participants should have received lectures or other instructions in outbreak investigations, including case definition development, line listing, passive and active case search to populate a line list, construction of an epi-curve and its interpretation, and report writing and information dissemination.

**Materials needed:** laptop with Microsoft Excel, Epi Info™, or graph paper; calculator; flipchart or whiteboard with markers; A4 sheets; pens; pencils; and erasers.

**Level of training and associated public health activity:** Novice - outbreak investigation

**Time required:** 2-3 hours

**Language:** English

## Competing interest

The authors declare no competing interest.
